# *Escherichia coli* Affects Expression of Circadian Clock Genes in Human Hepatoma Cells

**DOI:** 10.3390/microorganisms9040869

**Published:** 2021-04-17

**Authors:** Urša Kovač, Zala Žužek, Lucija Raspor Dall’Olio, Katka Pohar, Alojz Ihan, Miha Moškon, Damjana Rozman, Marjanca Starčič Erjavec

**Affiliations:** 1 Centre for Functional Genomics and Biochips, Faculty of Medicine, University of Ljubljana, 1000 Ljubljana, Slovenia; ursa.kovac@mf.uni-lj.si (U.K.); lucija.raspor-dallolio@mf.uni-lj.si (L.R.D.); damjana.rozman@mf.uni-lj.si (D.R.); 2 Biotechnical Faculty, University of Ljubljana, 1000 Ljubljana, Slovenia; zuzek.zala@gmail.com; 3 Institute of Microbiology and Immunology, University of Ljubljana, 1000 Ljubljana, Slovenia; katka.pohar@mf.uni-lj.si (K.P.); alojz.ihan@mf.uni-lj.si (A.I.); 4 Faculty of Computer and Information Science, University of Ljubljana, 1000 Ljubljana, Slovenia; miha.moskon@fri.uni-lj.si

**Keywords:** intestinal microbiota, *Escherichia coli*, circadian clock, circadian rhythm, HepG2

## Abstract

Recent research has indicated that dysbiosis of the gut microbiota can lead to an altered circadian clock of the mammalian host. Herein we developed an original system that allows real-time circadian studies of human HepG2 hepatoma cells co-cultured with bacteria. The HepG2 cells with stably integrated firefly luciferase reporter under the control of *PERIOD2* promoter were co-cultured with *E. coli* strains isolated from human fecal samples from healthy individuals. The two *E. coli* strains differ in the phylogenetic group and the number of ExPEC virulence-associated genes: BJ17 has only two, and BJ23 has 15 of 23 tested. In the first 24 h, the *E. coli* BJ17 affected the HepG2 circadian clock more than BJ23. Cosinor analysis shows a statistically significant change in the amplitude of *PER1* and *2* and the phase advance of *PER3.* A high percentage of necrotic and apoptotic cells occurred at 72 h, while a correlation between the number of ExPEC genes and the influence on the HepG2 core clock gene expression was observed. Our study reveals that the *E. coli* genetic background is important for the effect on the mammalian circadian clock genes, indicating possible future use of probiotic *E. coli* strains to influence the host circadian clock.

## 1. Introduction

Gut microbiota has been extensively studied in recent years, and multiple features associated with human health have been discovered. It is now clear that the bacteria in our intestine impact host metabolism [1,2], prevent colonization by pathogens [3], affect the immune system [4,5] and through all these actions help to maintain the homeostasis of the host. An “imbalance” of the microbiota, the so-called “dysbiosis”, has been associated with different diseases, ranging from bowel diseases to psychiatric disorders [6]. Moreover, the gut microbiota interferes with the circadian clock of the host [1,2,7,8,9,10]. While it became accepted that microbiota can modulate the host’s core clockwork machinery, we know very little about the influence of individual bacterial strains.

The circadian system consists of the central circadian clock (=central pacemaker) located in the hypothalamus, more precisely in the suprachiasmatic nucleus (SCN), and peripheral clocks in almost every mammal cell and tissue. There are many cues, such as light, food intake, metabolites and sleep cycle that orchestrate the circadian system. On the molecular level, circadian rhythms are maintained by two interlocking transcription–translation feedback loops. The core loop consists of transcriptional activators circadian locomotor output cycles kaput (*CLOCK*), brain and muscle Arnt-like protein 1 (*BMAL1*), and transcriptional repressors period (*PER1*, *PER2* and *PER3*) and cryptochrome (*CRY1* and *CRY2*) [11]. Furthermore, kinases and phosphatases play an important role for localization and stability of these integral clock proteins. The second loop represents transcriptional activators retinoid related orphan receptors (*RORa*, *RORb*, *RORc*) and repressors *REVERBa/REV-ERBb* [12].

Changes in composition and function of intestinal microbiota and microbial metabolites, such as vitamins, amines, butyrate and polyphenolic derivatives, can alter the host circadian rhythms [2,5,7,8,9,10]. Dysbiosis of gut microbiota affects the circadian clock gene transcription as well as the host metabolism. For example, germ-free mice have an impaired central and hepatic circadian clock gene expression [1,8], underlining the role of the host microbiome. However, there is little information about the role of individual bacterial species, among them *Escherichia coli*, in influencing the circadian clock. *E. coli* is known to be genetically very diverse. According to the relationship of *E. coli* with its host, we distinguish between commensal, extraintestinal pathogenic (ExPEC) and intestinal pathogenic (IPEC) *E. coli* strains. Commensal *E. coli* inhabit the human intestine within hours after birth and stay for decades as a part of normal microbiota [13]. However, ExPEC strains are also found among the gut microbiota, but they have obtained multiple virulence factors, which allow them to cause diseases. When ExPEC are transmitted to non-intestinal anatomic sites, such as the urinary tract, they instigate infection [14,15]. On the other hand, IPEC strains are not part of the gut microbiota. After the consumption of drinking water contaminated with IPEC or of contaminated food, they typically cause diarrheal disease of the intestinal tract [13,16].

The liver provides the first line of defense against microbes crossing the intestinal barrier [17]. This can be considered as the intravascular firewall that captures and eliminates pathogens from the blood [18]. In the healthy state, lipopolysaccharide (LPS), flagellin and other products of microbiota penetrate the intestinal wall only in trace amounts. The gut-derived LPS plays a central role in the induction of liver pathologies [19]. When the intestinal barrier is disrupted, which happens often in the case of alcoholic [20] and non-alcoholic [21] liver disease, the bacterial translocation to the liver is accelerated. In such a case, the gut products can come to the liver and initiate the liver disease known as MAFLD (metabolic associated liver disease), which can progress through metabolic associated steatohepatitis (MASH) towards the terminal stage of hepatocellular carcinoma [22,23,24]. In comparison to healthy subjects, the MAFLD patients show increased amounts of some bacterial species, including *E. coli* [25,26]. Intestine permeability can temporarily increase also for non-pathologic reasons such as endurance exercise, non-steroidal anti-inflammatory drugs administration, pregnancy and surfactants (such as bile acids and dietary factors such as emulsifiers) [27]. The translocation of bacterial products and, in the case of a disrupted epithelial barrier, vial gut bacteria, such as *E. coli* [25], from the intestine to the liver also induces inflammation in Kupffer cells, a fibrotic response of hepatic stellate cells, with damaging effects on the hepatocytes [28]. The role of Kupffer cells is to clear the microorganisms from the circulation, while hepatocytes secrete cytokines and chemokines in response to bacterial invasion [26,29]. A response to bacterial invasion might also be a change in the circadian clock of the hepatic cells. Human hepatoma HepG2 cells have been used as a valuable in vitro cell model, which has enabled the study of how different factors [30,31], including bacterial infection [32], affect circadian rhythm and its key genes [33,34] in the liver.

Herein, we investigated the *E. coli* isolated from fecal samples obtained from healthy individuals in co-culture with human hepatoma HepG2 cells. HepG2 cells are a suitable in vitro model system for the study of polarized human hepatocytes. These cells can activate innate immunity against invading microorganisms by secreting innate immunity proteins [35] and have been used in studying the anti-inflammatory effect of lactobacilli bacteria [36]. In our study, we focus on two *E. coli* strains, one with many ExPEC virulence-associated genes and another with only a few such genes, in light of their potential to interfere with the mammalian hepatic circadian clock.

## 2. Materials and Methods

### 2.1. Data and Code Availability

The qPCR data are available via https://data.mendeley.com/datasets/pjbyxys4mw/draft?a=43f48715-2c44-46ff-8baa-713e3351e95c (accessed on 15 April 2021).

### 2.2. Experimental Model and Subject Details

Preparation of Human Hepatoma cells with stably integrated *PER2-dluc*.

HepG2 cells were obtained from ATCC (HB-065) and kept stored in liquid nitrogen until used. The cells were cultured in a monolayer in Dulbecco’s Modification of Eagle’s Medium (DMEM, Sigma Aldrich, Taufkirchen, Germany, SI-D6429) supplemented with a 10% fetal bovine serum (FBS, Sigma Aldrich) and 1% penicillin/streptomycin (PS, Sigma Aldrich) antibiotic mix at 37 °C in an atmosphere of 5% CO_2_. When the cells reached 70–80% confluency, they were trypsinized, resuspended in fresh media and used in further experiments.

To enable real-time monitoring of circadian cell behavior with the LumiCycle apparatus 32 (Actimetrics, Wilmette, IL, USA), a new stable cell line transfected with *PER2-dluc* plasmid was prepared. PolyJet™ In Vitro DNA Transfection Reagent (SignaGen, Frederick, MD, USA) and the PolyJet^TM^ advanced protocol for transfecting mammalian cells was used for stable introduction of *PERIOD2* promoter controlling the Luciferase reporter (*PER2-Luc*) into the genome of HepG2 cells. The protocols were performed according to the manufacturer’s instructions. Further, the selection of antibiotic-resistant clones and single cell cloning was performed in 96 well plates. Clones with the best circadian parameter characteristics as determined on LumiCycle were chosen for further analysis.

### 2.3. Microbial Strains

Two *E. coli* strains from the BJ collection of *E. coli* strains isolated from fecal samples of healthy individuals [37,38], BJ17 and BJ23, were chosen based on the previously characterized strain’s ExPEC virulence-associated genotype. BJ17 belongs to the phylogenetic group A and has the following ExPEC virulence-associated genotype: *papGII–*, *papGIII*–, *afa/dra*–, *sfaDE*–, *iha*–, *fimH*–, *ibeA*–, *fyuA*+, *hbp*–, *ireA*–, *iucD*–, *iroN*+, *hlyA*–, *usp*–, *clbAQ*–, *picU*–, *kpsMTII*–, *ompT*–, ompT_APEC_–, *tcpC*–, *iss*–, *neuB*–, *traT*–. BJ23 belongs to the phylogenetic group B2 and has the following ExPEC virulence-associated genotype: *papGII–*, *papGIII*–, *afa/dra*–, *sfaDE*+, *iha*–, *fimH*+, *ibeA*+, *fyuA*+, *hbp*+, *ireA*+, *iucD*+, *iroN*+, *hlyA*–, *usp*–, *clbAQ+*, *picU*–, *kpsMTII*+, *ompT*+, ompT_APEC_+, *tcpC*–, *iss*+, *neuB*+, *traT*+. Strains were transferred from their storage at −80 °C and grown on Luria Bertani (LB, Biolife) agar or broth at 37 °C; in case of growth in broth, the culture was aerated (180 rpm). When the bacterial strains were in co-culture with the HepG-2 cells, they were grown in DMEM. For use, the bacterial strains were stored on LB agar at 4 °C for up to 3 weeks, then transferred from their storage at −80 °C again.

### 2.4. Method Details

#### 2.4.1. Bacterial Growth Curves

For the determination of bacterial growth curves, bacterial strains were grown in liquid LB media overnight at 37 °C with aeration (5% CO_2_). The next day, overnight cultures were diluted 1:100 in fresh, pre-warmed LB media in a conical flask and grown for 7 h. Each hour, samples were taken for measurement of optical density at 600 nm (OD600) and for determination of colony-forming units per milliliter (CFU/mL) on LB plates. Before transfer onto the LB plate, samples were 10-fold serial diluted (Table S24) in PBS. Data were obtained from two independent experiments performed in duplicate.

Moreover, the data obtained in these growth curve experiments were used in linear regression analysis (Figure S1B) to estimate the relationship between OD600 and CFU/mL in order to be able to calculate the needed amount of bacteria for multiplicity of infection (MOI) 0.005:1 (bacteria:HepG2 cell) in co-culture experiments with HepG2 cell line.

#### 2.4.2. Stable Co-Culture Preparation

Bacterial strains were transferred from LB plates stored at 4 °C to LB media and grown overnight at 37 °C and 5% CO_2_. The next day, bacterial cultures were diluted 1:100 in fresh, pre-warmed LB media and incubated for 2.5–3 h to reach the early exponential phase. Based on the OD600 measurements, the needed amount of bacteria for MOI 0.005 was resuspended in recording media (200 µM L-glutamine (Gibco, Waltham, MA, USA), 100 µM Na-pyruvate (Gibco, Waltham, MA, USA), 10% FBS, 4 % d-luciferin (Sigma Aldrich, Taufkirchen, Germany) and DMEM: high glucose, no glutamine, no phenol red) and transferred onto synchronized HepG2 *PER2-dluc* cells cultured on 34 mm dishes. Dishes were sealed with parafilm and placed in LumiCycle apparatus for 24 h (37 °C, 5% CO_2_). Each experiment was performed in duplicate on two 35 mm Petri dishes.

#### 2.4.3. 72 h Sampling

Bacterial cultures used to infect the HepG2 cells were prepared as described in Stable co-culture preparation, with the exception that they were finally resuspended in DMEM supplemented with 10% FBS. The bacterial suspension was then transferred on synchronized HepG2 cells cultured in 24-well plates. Co-culture was incubated for 4 h. Then, bacteria were discarded and cells were washed 3 times with PBS before DMEM supplemented with a 10% FBS and 1% penicillin/streptomycin antibiotic mix was applied. Timepoint 0 was determined as the moment when bacteria were discarded. Sampling was performed every 2 h for 24 h, with additional time points at 48 h and 72 h. Samples, obtained in two independent experiments—two separated wells on the same plate and performed in triplicate—were stored at −80 °C until the isolation of total RNA.

#### 2.4.4. RNA Isolation and RT-PCR Analysis

RNA from HepG2 cells was extracted by Trizol reagent (Sigma Aldrich) following manufacturer’s instructions. Quality and quantity of RNA were determined using NanoDrop™ (ThermoFisher Scientific, Waltham, MA, USA), diluted accordingly and after DNA removal with DNase I Recombinant Kit (Roche, Basel, Switzerland) transcribed to cDNA with Transcriptor Universal cDNA Master (Roche) according to the manufacturer’s protocols. PCR analysis was performed on Lightcycler 480 using Sybr Green Master Mix (Roche, Basel, Switzerland) kit according to manufacturer’s protocol. Primers used are listed in Table S25. Target gene expression was normalized on reference genes *Rplp0* and *ActB*, appraising also amplification efficiency of a qPCR.

#### 2.4.5. Apoptosis-Necrosis Assay

For the apoptosis-necrosis assay, FITC Annexin V Apoptosis Detection Kit II (cat. num. 556570, BD Pharmigen) was used. Time points 0, 24, 48 and 72 h were chosen for this assay. Non-treated cells were used as negative control. After incubation (0, 24, 48 or 72 h), cells were washed with PBS and harvested by trypsinization (Trypsin (Sigma Aldrich), 1 min. incubation), centrifuged at 500× *g* for 5 min, and washed with 1% BSA (Sigma Aldrich) in PBS. Then, a sample for flow cytometry analysis was prepared according to manufacturer protocol. Each sample was filtered through polystyrene tube with strained cap and analyzed by the machine BD FACSCanto II System (BD Biosciences, Franklin Lakes, NJ, USA) with the BD FACSDiva™ Software (BD Biosciences, Franklin Lakes, NJ, USA). The assay was performed in duplicates.

#### 2.4.6. Quantification and Statistical Analysis

Microsoft Excel was used for calculation of the mean ±SD and to perform the linear regression analysis. For comparison analysis of circadian genes expression at time points 24 h, 48 h and 72 h, GraphPad PRISM (v6.01, GraphPad Software, San Diego, CA, USA) was used (Sidak’s comparison test).

Rhythmicity analysis of observed circadian genes was performed with a single-component cosinor model [39], for which a 24 h period was presumed. This model can be described as

(1) $$Y=\alpha \cdot X_{1}+\beta \cdot X_{2}+\gamma ,$$

where $X_{1}=sin\left(\frac{T}{24}\cdot 2\pi \right)$ and $X_{2}=cos\left(\frac{T}{24}\cdot 2\pi \right)$, and where T presents the vector of time points in which measurements were obtained. We fitted the measured data to this model using linear regression. The obtained models were used to assess the circadian parameters of the data and their significance. We evaluated the amplitudes and acrophases using Equations (2) and (3), respectively.

(2) $$A=\sqrt{\alpha^{2}+\beta^{2}}$$

(3) $$\theta =\{\begin{array}{cc} -atan\left(\frac{\alpha }{\beta }\right);\alpha >0, & \beta >0\\ -1+atan\left(-\frac{\alpha }{\beta }\right);\alpha >0, & \beta <0\\ -2\pi +atan\left(\frac{\alpha }{\beta }\right);\alpha <0, & \beta >0\\ -2\pi -atan\left(\frac{\alpha }{\beta }\right);\alpha <0, & \beta <0 \end{array}.$$

We evaluated the significance of rhythmicity of fitted data with zero-amplitude test using the F-statistic as described in Bingham [40].

An extended single-component cosinor model can be used to assess the differential rhythmicity among pairs of different circadian experiments:

(4) $$Y=\left(\alpha_{1}+g\cdot \alpha_{2}\right)\cdot X_{1}+\left(\beta_{1}+g\cdot \beta_{2}\right)\cdot X_{2}+\gamma ,$$

where parameter g equals 0 if the data belong to the first experiment, and 1 if the data belong to the second experiment, respectively. We applied this model to different pairs of experiments, namely control (K) vs. perturbation (BJ17 or BJ23), to assess the difference of circadian parameters between the experiments (amplitude change and phase shift) as well as their significance [40].

Rhythmicity and differential rhythmicity analyses were implemented in Python 3 using a recently developed CosinorPy package [41]. The reported significance values are adjusted for multiple testing using the Benjamini and Hochberg procedure.

## 3. Results

### 3.1. Assessing Growth Curves of E. coli Strains BJ17 and BJ23

The characteristics of both *E. coli* bacterial strains are described in the Microbial strains section. Growth characteristics of BJ17 strain (low number of ExPEC virulence-associated genes) and BJ23 strain (high number of ExPEC virulence-associated genes) were evaluated during a 7 h incubation as described in the Bacterial Growth Curves section. The results (Figure S1A) show similar growth curves for both bacterial strains, as determined by measurement of optical density at 600 nm (OD600) and determination of colony-forming units per milliliter (CFU/mL) on LB plates. The bacterial growth data obtained in the exponential phase (2–5 h) were analyzed with linear regression (Figure S1B). The obtained ratio OD600 to CFU/mL was used in further experiments to determine the needed amount of bacteria for multiplicity of infection (MOI) 0.005 in co-culture.

### 3.2. Co-Culture of HepG2 Cells with Bacterial Strains Is Stable for 4 h

**Figure 1:**
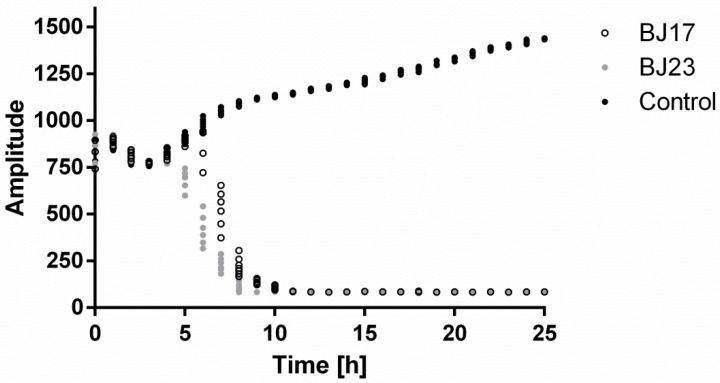
HepG2 *PER2-dluc* cell line activity measured with Lumicycle. Co-cultures with bacterial strain BJ17 (white dots) or BJ23 (grey dots) and control HepG2 *PER2*-*dluc* cells without bacteria (black dots). Data are represented as the mean of two separate experiments.

HepG2 cells with stably integrated firefly luciferase reporter under control of the *PERIOD2* promoter (HepG2 *PER2-dluc*) were inoculated with either BJ17 or BJ23 *E. coli* strain, and their co-culture was followed for 25 h in real time with the Lumicycle apparatus. As seen in Figure 1, after 4 h, the signal of the measured luminescence in co-cultures decreased, while the luminescence signal of the control, HepG2 *PER2-dluc* cells without bacteria, was still increasing. From this, we concluded that both co-cultures are stable for 4 h, and therefore, in all following experiments, the incubation of the co-cultures was always 4 h.

### 3.3. The 4 h Incubation of HepG2 Cells with E. coli Affects Expression of HepG2 Circadian Genes

**Figure 2:**
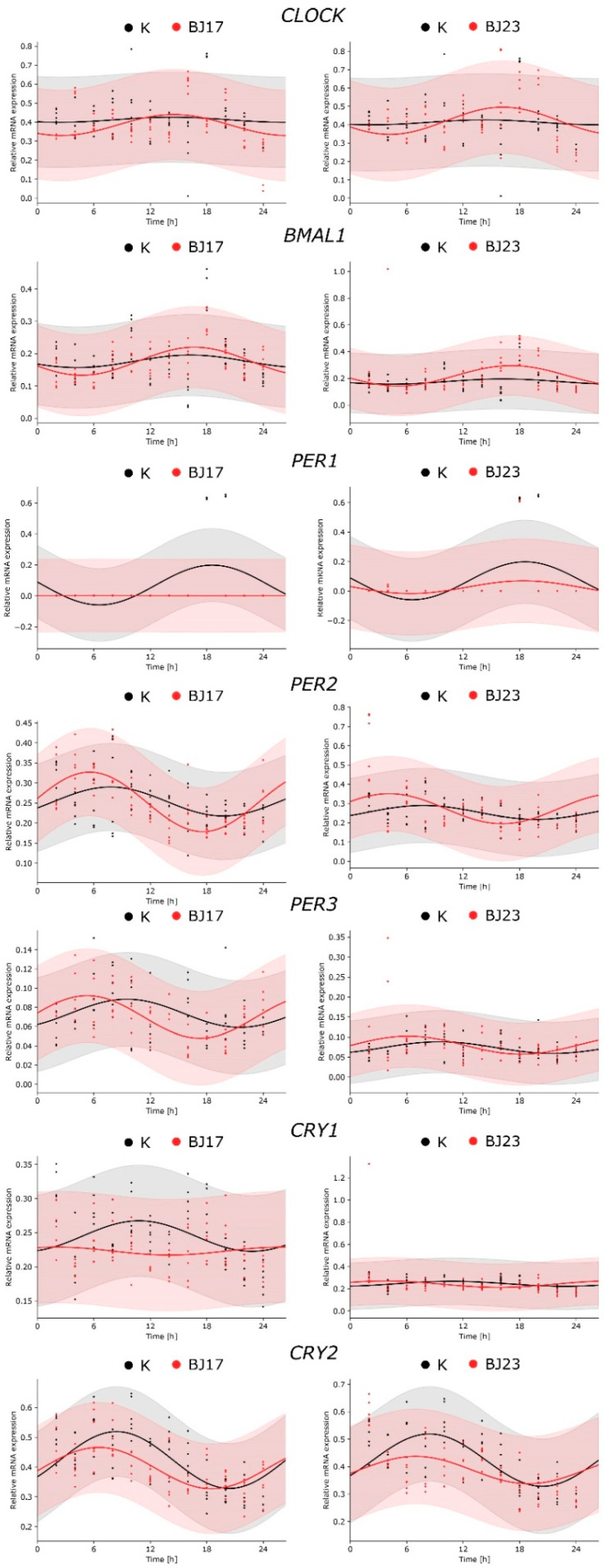
Comparison of circadian behavior of core clock gene expression between the co-cultures and control. The figures represent the relative RNA expression of the core clock genes as a function of time (2–24 h) in HepG2 cells (K, black dots and lines, respectively) and in HepG2 cell co-cultures with *E. coli* strains BJ17 and BJ23 (red dots and lines, respectively). The experimental results, obtained in two separate experiments, are represented with dots, and the fitted cosinor curves with solid lines. The raw data used in the fitting process are available in Tables S2–S22. The quantitative results obtained with the cosinor analysis are available in Tables S1 and S23.

After the 4 h incubation of HepG2 cells with bacterial strains, the bacteria were removed, and the HepG2 cells were prepared for the 72 h sampling assay in order to reveal the influence of applied *E. coli* on the hepatic HepG2 circadian clock. HepG2 cells were sampled every 2 h during the first 24 h and then two more samples were collected, one at 48 h and the other at 72 h. The central genes of the circadian clock, transcriptional activators *CLOCK* and *BMAL1*, and repressors *PER1*, *PER2*, *PER3*, *CRY1* and *CRY2* were studied and statistical analyses were performed. The first 24 h were analyzed with the cosinor test. The results in Figure 2 show the core clock gene expression in HepG2 cells grown in the absence (control—K) or presence of *E. coli* strains BJ17 and BJ23. In control HepG2 cells, the statistical tests (Table S1) confirmed the circadian expression of the genes encoding circadian repressors *PER1,2,3* and *CRY1,2,* while we failed to detect the circadian wave for the activator proteins *CLOCK* and *BMAL1.* The addition of bacteria retained the rhythmic expression of HepG2 core clock genes except for *CRY1* and enhanced the circadian behavior of *CLOCK* and *BMAL1.* Taken together, the measured core clock genes, with the exception of *CRY1*, were expressed rhythmically also after being exposed to *E. coli* strains.

An important aspect in evaluating whether bacteria affected the inner clock of mammalian cells is the estimation of circadian parameters (Table S23). A statistically significant change of the amplitude, compared to controls, was observed for HepG2 genes *PER1* (decrease) and *PER2* (increase) grown with *E. coli* BJ17. The change in the phase of gene expression (phase shift) was observed only for *PER3*, again grown with *E. coli* BJ17. The rhythmic expression of *PER3* was advanced by 4.23 h, meaning that the expression of this circadian repressor was phase-advanced compared to other core clock genes.

**Figure 3:**
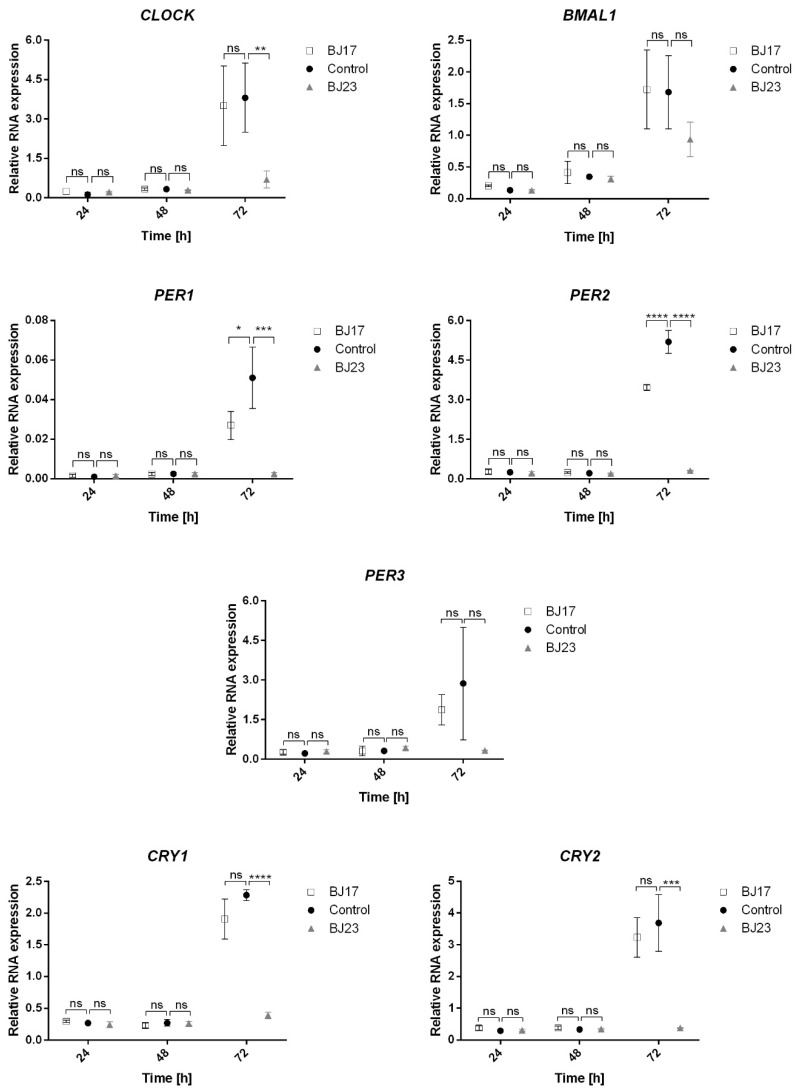
Relative expression of the core circadian genes as a function of time (24–72 h). BJ17 (white squares), BJ23 (grey triangles): co-cultures of BJ strain with the HepG2 cells. Control (black circles): HepG2 cells without bacteria. Statistically significant results were presented; not significant (ns) for *p* > 0.05, * for *p* ≤ 0.05, ** for *p* ≤ 0.01, *** for *p* ≤ 0.001 and **** for *p* ≤ 0.0001. Data are represented as mean ± SD of two separate experiments.

To evaluate whether the 4 h co-culture of the *E. coli* strains affects the long-term expression of the core clock genes, we monitored their expression at 24 h, 48 h and 72 h (Figure 3). It is evident that both *E. coli* strains diminish the expression of the core clock genes after 72 h. The relation between the number of ExPEC virulence-associated genes and the influence on the circadian clock of HepG2 cells is obvious. The cells co-cultured with *E. coli* BJ23 with more virulence factors in most cases show a lower expression of core clock genes compared to cells co-cultured with *E. coli* BJ17. Exceptions are the transcriptional activator *BMAL1* and the repressor *PER3*, where there is no statistical difference between the bacterial strains.

### 3.4. The 4 h Incubation of HepG2 Cells with the Bacterial Strain with More Virulence-Associated Genes Did Affect the HepG2 Cells Viability

**Figure 4:**
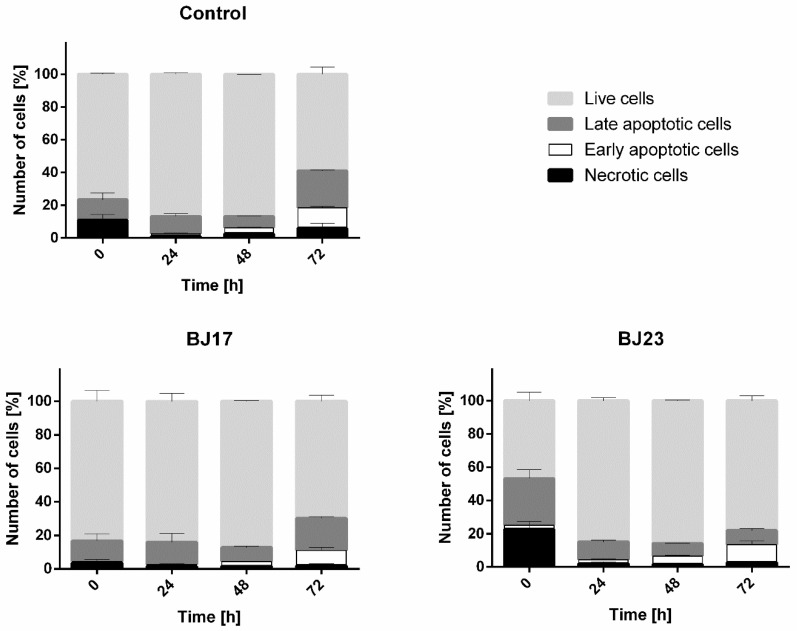
Results of apoptosis/necrosis test. Percentages of live cells (light grey), late apoptotic cells (dark grey), early apoptotic (white) and necrotic (black) cells are represented as mean ± SD of two separate experiments.

The viability of HepG2 cells was determined by the apoptosis/necrosis assay and flow cytometry. After the 4 h incubation of HepG2 cells with bacterial strains, the bacteria were removed and the apoptosis/necrosis assay on HepG2 cells was performed at 0 h (immediately after removal of the bacteria), 24 h, 48 h and 72 h after bacterial removal. The results (Figure 4) showed that more HepG2 cells were apoptotic and necrotic in co-culture with *E. coli* strain BJ23 possessing more ExPEC virulence-associated genes. Importantly, the viability of HepG2 cells in the co-culture with BJ17 strain was similar to controls, where HepG2 cells were grown without bacteria (Figure 4). Figure 4 shows also that the largest percentage of apoptotic and necrotic cells in both co-cultures was at time point 0 h.

## 4. Discussion

The circadian clock was most recently recognized as the essential molecular link between the physiology of the mammalian host and microorganisms. There is more evidence that the host mammalian circadian clock shapes the microbiome [42]. However, data about bidirectional communication between microorganisms and their hosts is increasing [5]. We are only in the early phases of understanding how the microbiota, and its metabolic outputs affect the rhythmic processes, such as immunity [42]. Microbiota can influence the host’s circadian gene expression in peripheral tissues by the produced metabolites, such as short-chain fatty acids and unconjugated bile acids [43]. As most results regarding the host–microbiome circadian interactions originate from mouse models, it is also essential to obtain verification results in human-derived cells and tissues.

Herein we developed an original system that allows real-time circadian studies of human hepatoma cells co-cultured with bacteria. Hepatocytes are key cell types for innate immunity [35] and therefore they represent an interesting model to study bacteria-human host interaction. Cell line HepG2 has been used in previous studies to demonstrate the preventive effect of drug candidates on circadian rhythm disorders [30,31]. The HepG2 cells with stably integrated firefly luciferase reported under the *PERIOD2* promoter were co-cultured with two *E. coli* strains isolated from human fecal samples obtained from healthy individuals. These bacterial strains differ in the phylogenetic group and number of present ExPEC virulence-associated genes. The BJ17 strain can be designated as a commensal *E. coli* strain as it belongs to the A phylogenetic group, which is associated with commensal strains [15] and has only two of the tested ExPEC virulence-associated genes, both belonging to the iron uptake systems (*fyuA* of the yersiniabactin and *iroN* of the salmochelin iron uptake system). The BJ23 strain can be designated as a potential ExPEC strain, as it belongs to the phylogenetic group B2, which is associated with ExPEC strains [15] and possesses 15 out of 23 tested ExPEC virulence-associated genes, including the *ibeA* encoding the invasin IbeA and *clbAQ* associated with the genotoxin colibactin. Based on the results obtained from the Lumicycle experiment (Figure 1), it can be concluded that HepG2 co-cultures with both *E. coli* strains (BJ17-HepG2 *PER2-dluc* and BJ23-HepG2 *PER2-dluc*) are stable for 4 h.

We were interested to examine whether the HepG2 core circadian clock was influenced by the bacteria in line with their number of ExPEC virulence-associated genes. We tested the hypothesis of whether bacteria with more ExPEC virulence-associated genes have a stronger effect on the host’s circadian clock. A simple statistical analysis of the core clock gene expression at 24 h, 48 h and 72 h showed that, only after 72 h, the relation between the number of ExPEC virulence-associated genes and the influence on the core circadian clock gene expression of HepG2 cells can be confirmed (Figure 3). In the first 24 h after co-culture of HepG2 cells with *E. coli*, the *E. coli* BJ17 seems to affect the circadian clock more than *E. coli* BJ23 (Figure 2). The cosinor analysis with adjusted *p*-value shows a statistically significant change in the amplitude of *PER1* and *PER2*, and the phase advance of *PER3,* all in the case when HepG2 cells were co-cultured with the less virulent *E. coli* BJ17. In the literature, we can see that pathogen-associated molecular patterns caused several modulations of the clock gene expression. In particular, researchers showed that the synthetic triacylated lipopeptide Pam3CSK4, and the ODN 1826, a DNA rich with CpG motifs as it is common for bacterial DNA, caused increased mRNA levels of *PER2* [10]. Likewise, we could observe an increase in the *PER2* amplitude of HepG2 in contact with both strains; in the case of BJ17, the increase was found to also be statistically significant. Literature reports that certain bacteria can also affect *Bmal1* expression: *Helicobacter pylori* activates transcription of *LIN28A* in gastric tissue, which binds to the *BMAL1* promotor. In this way, *H. pylori* infection upregulates the transcription of *BMAL1* and dysregulates the molecular clock in gastric tissues. Enhanced levels of *BMAL1* stimulate the expression of pro-inflammatory cytokine TNF-α and promote inflammation [44]. On contrary, *BMAL1* was downregulated in hepatocytes at *Staphylococcus oralis* infection, and it affected also coagulation processes in the liver [32]. In our case hepatocytes co-cultured with *E. coli* enhanced amplitude of expression of *BMAL1* compared to control, although not significantly. Thus, it would be tempting to speculate that bacterial infection affects the expression of circadian genes, but reaction depends on host tissue and the properties of bacteria, but, so far, this hypothesis has not been tested.

In addition, LPS, an endotoxin commonly found in the outer membrane of Gram-negative bacteria, has been shown to modify circadian clock genes. LPS changed the expression of core circadian genes and linked immune pathways. LPS injection suppressed expression of circadian genes: *Per1*, *Per2*, *Dbp*, *PPARα*, and *rFKBP51* mRNA in rats liver [45]; *Per2* in mouse ovary [46], in macrophages [47] and synovial cells [48]; *Per1* and *Per2* in hearth and liver [49]. Based on these findings, *Per2* appears to be suppressed by LPS immune response linked to circadian genes, and *PER2* was enhanced, likely due to the use of whole bacteria and not just LPS as in other studies mentioned above. In addition, we expected that TLR4—an important transmembrane immune receptor for LPS and FimH adhesin [50]—would be an important player in bacteria–hepatocytes contact. We thus monitored TLR4 expression initially 24 h after co-culture, and its expression was not rhythmic’ neither significantly differed between HepG2 cells in co-culture with *E. coli* BJ17, BJ23 compared to control without bacteria (see Figure S3). All this information suggests that LPS is probably not a key player in measured variations of circadian clock changes.

The observed amplitude changes of *PER1, PER2* and the phase advance of *PER3* can be orchestrated by the host innate immune response. Hepatocytes react to bacterial invasion by increasing the levels of pro-inflammatory factors IFN, IL-1β, and TNF-α. If we look closer at how these immune factors affect circadian genes, we can see that IL-1β attenuates *PER3* expression and TNF-α downregulates the rhythmic expression of *PER1, 2* and *3* [51]. TNF-α does not decrease the expression of *BMAL1*, but it inhibits the CLOCK/BMAL1 transactivation E-box in the *Per* and *Cry* promotors, which results in the decrease in its expression [52]. As an important mediator in the defense against invading pathogens, TNF-α has been induced in hepatocytes exposed to bacteria *Salmonella typhimurium* and *Listeria monocytogenes* [53]. TNF-α is probably present also in *E. coli* co-cultured HepG2 cells, although we were not able to detect it by qPCR due to the low quantity of available RNA.

The reasons for this result might be the short period of circadian sampling (only 24 h) and/or a high percentage of apoptotic HepG2 cells in co-culture with *E. coli* BJ23 at the start of the circadian sampling (Figure 4). The chosen *E. coli* strains were only tested for the most common ExPEC virulence-associated genes [15,54], but our results clearly demonstrate that *E. coli* BJ23, with more ExPEC virulence-associated genes, causes more HepG2 cells to enter apoptosis or necrosis compared to *E. coli* BJ17 at time 0 h (Figure 4). After 24 h and 48 h, most of the cells recovered, but a high percentage of necrotic and apoptotic cells occurred after 72 h. After three days, nutrient deprivation and cell density can affect the expression of housekeeping genes [55,56] and the relative expression may appear much higher. On the other hand, after 72 h, the percentage of live cells is at least 60%, and at the same time, instant the relative expression of certain circadian genes in HepG2 cells in co-culture with *E. coli* BJ23 is 3-5 times lower compared to control cells. It is hard to believe that this situation is a consequence of the high percentage of cell death, only, but more likely a consequence of active immune response. In fact, we measured the relative mRNA expression of *IL-6R*, receptor of multifunctional cytokine IL-6, which is induced by stimulation of IL-1, TNF-alpha or TLR-4, and expression changed significantly after 72 h (Figure S2 in the Supplementary Materials).

It was already shown that the circadian gene expression, particularly for the core clock activators *CLOCK* and *BMAL1,* is altered in apoptotic cells [57]. Although we cannot conclude that the number of ExPEC virulence-associated bacterial genes is the reason why bacterial strains altered the host’s circadian clock differently, our research anyway implies there is a connection that should be investigated further [55,56].

Whether and how individual virulence-associated gene affects the circadian rhythm of the host is still unknown. Knowledge of the ExPEC virulence-associated genes of bacterial strains in microbiota and their connection to the circadian clock could lead to the production of probiotics that would help the host to maintain the homeostasis of the circadian clock in the modern lifestyle (jet lag, shift work, late-night meals, etc.).

In conclusion, this study presents an original system that allows real-time circadian studies of human HepG2 hepatoma cells co-cultured with bacteria. The obtained data on this model revealed that the addition of bacteria retained the rhythmic expression of HepG2 core clock genes except *CRY1,* and enhanced the circadian behavior of *CLOCK* and *BMAL1.* Moreover, individual *E. coli* strains can have a different impact on the host circadian clock genes.

## Data Availability

All data generated or analysed during this study are included in this published article and its supplemental material.

## Supplementary Materials

The following are available online at https://www.mdpi.com/article/10.3390/microorganisms9040869/s1, Figure S1. Growth characteristics of bacterial strains BJ17 and BJ23, Figure S2: Relative expression of the *IL-6R* genes as a function of time (24–72 hours), Figure S3: Comparison of circadian behaviour of core clock gene expression between the co-cultures and control, Table S1. Estimation of circadian behaviour of core clock gene expression in HepG2 cells (K) and in co-culture with *E. coli* strains BJ17 and BJ23, related to Figure 2, Table S2: Relative mRNA expression clock—incubation with no bacteria, control, Table S3: Relative mRNA expression *CLOCK*—incubation with strain *E. coli*, strain B17, Table S4: Relative mRNA expression *CLOCK*—incubation with strain *E. coli*, strain B23, Table S5: Relative mRNA expression *BMAL1*—incubation with no bacteria, control, Table S6: Relative mRNA expression *BMAL1*—incubation with strain *E. coli*, strain B17, Table S7: Relative mRNA expression *BMAL1*—incubation with strain *E. coli*, strain B23, Table S8: Relative mRNA expression *PER1*—incubation with no bacteria, control, Table S9: Relative mRNA expression *PER1*—incubation with *E. coli*, strain BJ17, Table S10: Relative mRNA expression *PER1*—incubation with *E. coli*, strain BJ23, Table S11: Relative mRNA expression *PER2*—incubation with no bacteria, control, Table S12: Relative mRNA expression *PER2*—incubation with *E. coli*, strain BJ17, Table S13: Relative mRNA expression *PER2*—incubation with *E. coli*, strain BJ23, Table S14: Relative mRNA expression *PER3*—incubation with no bacteria, control, Table S15: Relative mRNA expression *PER3*—incubation with *E. coli*, strain BJ17, Table S16: Relative mRNA expression *PER3*—incubation with *E. coli*, strain BJ23, Table S17: Relative mRNA expression *CRY1*—incubation with no bacteria, control, Table S18: Relative mRNA expression *CRY1*—incubation with *E. coli*, strain BJ17, Table S19: Relative mRNA expression *CRY1*—incubation with *E. coli*, strain BJ23, Table S20: Relative mRNA expression *CRY2*—incubation with no bacteria, control, Table S21: Relative mRNA expression *CRY2*—incubation with *E. coli*, strain BJ17, Table S22: Relative mRNA expression *CRY2*—incubation with *E. coli*, strain BJ23, Table S23. Comparison of circadian behaviour of core clock gene expression in HepG2 cells (K) in co-culture with *E. coli* strains BJ17 and BJ23, related to Figure 2, Table S24. Final dilutions of the bacterial culture in growth curve experiments, related to Bacterial Growth Curves, Table S25. List of used primers for RT-PCR, related to RNA isolation and RT-PCR analysis.
