# Supplementary material for: Escherichia coli Affects Expression of Circadian Clock Genes in Human Hepatoma Cells
Source: Microorganisms. 2021 Apr 17;9(4):869. doi: 10.3390/microorganisms9040869 (PMC8073551; doi:10.3390/microorganisms9040869)
Supplement: Supplementary file 1 [file microorganisms-09-00869-s001.zip › microorganisms-1175120-supplementary.pdf]

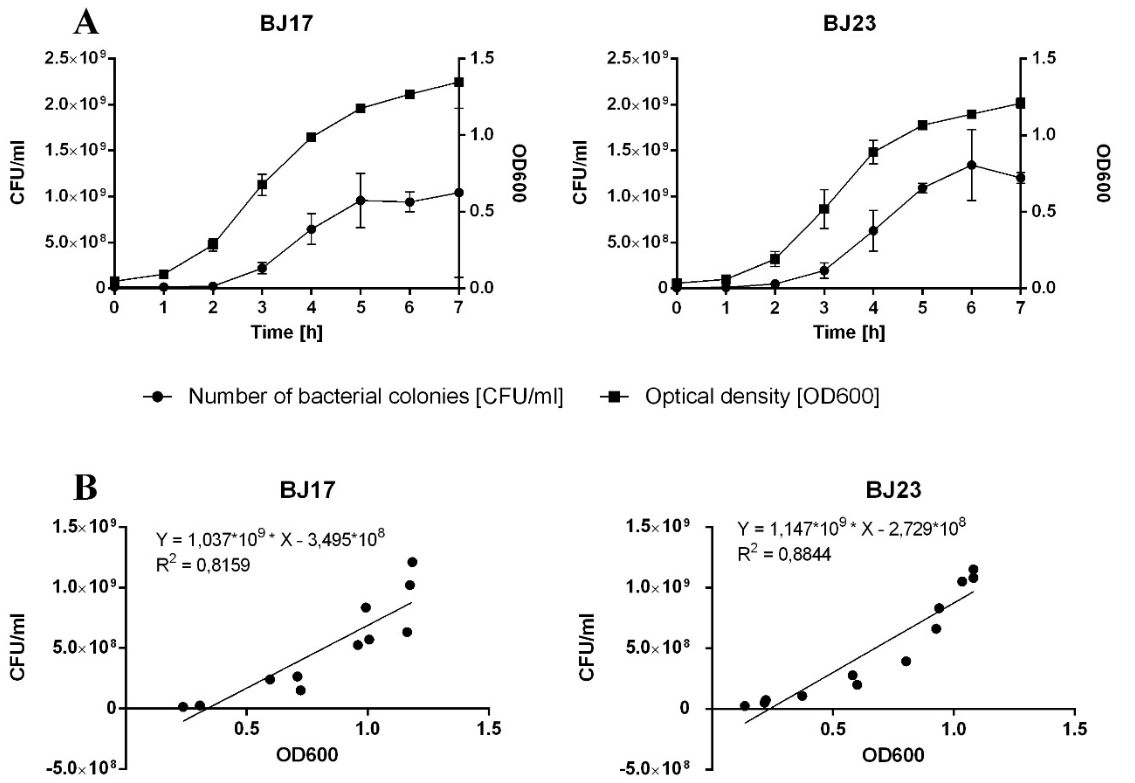

Figure S1. Growth characteristics of bacterial strains BJ17 and BJ23. (A) Bacterial growth curves. (B) Linear regression analysis of exponential phase data (2–5 hours). Data are represented as mean ± SD of three separate experiments.

Table S1. Estimation of circadian behaviour of core clock gene expression in HepG2 cells (K) and in co-culture with *E. coli* strains BJ17 and BJ23, related to Figure 2.

Gene	Circadian expression, adjusted p-value	Amplitude (a.u.)	Phase (radians)	Phase (hours)
CLOCK, K	5.40E-01	1.33E-02	-3.75	14.31
CLOCK, BJ17	6.28E-03	5.51E-02	-3.80	14.51
CLOCK, BJ23	1.77E-03	7.41E-02	-4.24	16.21
BMA1L, K	1.67E-01	1.94E-02	-4.26	16.26
BMAL1, BJ17	3.19E-08	4.33E-02	-4.37	16.70
BMAL1, BJ23	1.92E-03	7.61E-02	-4.48	17.12
PER1, K	4.36E-05	1.29E-01	-4.87	18.59
PER1, BJ17	4.47E-07	5.04E-04	-1.68	6.42
PER1, BJ23	3.60E-02	4.32E-02	-4.82	18.40
PER2, K	3.40E-04	3.62E-02	-2.03	7.76
PER2, BJ17	6.83E-17	7.43E-02	-1.46	5.56
PER2, BJ23	8.70E-04	7.81E-02	-1.08	4.14
PER3, K	4.21E-03	1.47E-02	-2.49	9.52
PER3, BJ17	9.26E-09	2.21E-02	-1.39	5.29
PER3, BJ23	7.25E-03	2.26E-02	-1.61	6.15
CRY1, K	6.56E-03	2.23E-02	-2.83	10.80
CRY1, BJ17	2.94E-01	5.84E-03	-0.53	2.04
CRY1, BJ23	2.65E-01	2.89E-02	-0.99	3.77
CRY2, K	4.50E-11	9.54E-02	-2.20	8.41
CRY2, BJ17	5.22E-09	6.95E-02	-1.71	6.51
CRY2, BJ23	1.92E-03	4.99E-02	-1.82	6.95

Table S2–S22: Relative mRNA expression of circadian behaviour of core clock genes in HepG2 cells (K) and in co-culture with *E. coli* strains BJ17 and BJ23, related to Figure 2 - **raw data**

Table S2: **Relative** mRNA expression *clock* – incubation with no bacteria, control

***clock* control**	Well A			Well B		
hours	Replicate 1	Replicate 2	Replicate 3	Replicate 1	Replicate 2	Replicate 3
2	0.422387	0.392902	0.403362	0.467188	0.448236	0.472457
4	0.381177	0.332871	0.316761	0.530632	0.33194	0.314997
6	0.459566	0.383735	0.324546	0.395601	0.384138	0.485194
8	0.50418	0.523615	0.564894	0.32261	0.338598	0.312307
10	0.504216	0.784092	0.517656	0.359779	0.390347	0.423207
12	0.510947	0.415249	0.46425	0.285852	0.286283	0.297331
14	0.389164	0.450306	0.440493	0.421263	0.39702	0.384592
16	0.294027	0.236633	0.011974	0.39684	0.380492	0.401375
18	0.760898	0.742336	0.74813	1.048965	1.031132	1.036322
20	0.431211	0.473302	0.459587	0.370522	0.404622	0.380145
22	0.398001	0.386877	0.367508	0.447372	0.384023	0.428886
24	0.292984	0.264447	0.266075			

Table S3: Relative mRNA expression *clock* – incubation with strain *E. coli*, strain B17

***clock* BJ17**	Well A			Well B		
hours	Replicate 1	Replicate 2	Replicate 3	Replicate 1	Replicate 2	Replicate 3
2	0.330071	0.312991	0.336311	0.937829	0.919272	0.932068
4	0.350724	0.327735	0.323544	0.561455	0.581052	0.550565
6	0.381095	0.365654	0.352533	0.360854	0.360606	0.446238
8	0.465115	0.463896	0.422587	0.334184	0.320155	0.444272
10	0.354569	0.294676	0.36583	0.328063	0.351295	0.376766
12	0.399603	0.362115	0.31383	0.402698	0.406325	0.374412
14	0.409758	0.347361	0.342919	0.297706	0.318788	0.334457
16	0.623037	0.667107	0.618386	0.58969	0.633238	0.549457
18	0.389792	0.415993	0.41762	0.346656	0.406878	0.378271
20	0.406053	0.382998	0.406604	0.555531	0.574475	0.516665
22	0.355837	0.302223	0.394123	0.272366	0.264955	0.264261
24	0.30915	0.250274	0.292259	0.281921	0.038956	0.069123

Table S4: Relative mRNA expression *clock* – incubation with strain *E. coli*, strain B23

***clock* BJ23**	Well A			Well B		
hours	Replicate 1	Replicate 2	Replicate 3	Replicate 1	Replicate 2	Replicate 3
2	0.409358	0.398961	0.374119	0.397753	0.393862	0.376401
4	5.101115	7.977533	6.394679	0.418145	0.483566	0.436411
6	0.417124	0.445028	0.485181	0.302744	0.298944	0.323309
8	0.458038	0.502327	0.496864	0.456736	0.395534	0.305599
10	0.386975	0.358505	0.384715	0.279568	0.268195	0.2612
12	0.448449	0.529643	0.560222	0.418286	0.488682	0.434331
14	0.391689	0.357447	0.405377	0.372622	0.387153	0.417929
16	0.465656	0.519313	0.397148	0.81128	0.805247	0.217799
18	0.48845	0.494409	0.467241	0.643749	0.687552	0.597383
20				0.617927	0.652642	0.696549
22	0.281186	0.251857	0.265803	0.333458	0.314932	0.333293
24	0.246896	0.202334	0.233399			

Table S5: Relative mRNA expression *bmal1* – incubation with no bacteria, control

***bmal1* control**	Well A			Well B		
hours	Replicate 1	Replicate 2	Replicate 3	Replicate 1	Replicate 2	Replicate 3
2	0.094991	0.131731	0.1873	0.215675	0.236206	0.2234314
4	0.229104	0.104196	0.179242	0.111805	0.135622	0.1225783
6	0.092877	0.143653	0.15826	0.193018	0.162103	0.1419226
8	0.196833	0.182356	0.152699	0.126787	0.138904	0.1242372
10	0.182859	0.306266	0.271342	0.278802	0.197173	0.318717
12	0.214524	0.215092	0.180132	0.12253	0.102999	0.106997
14	0.170558	0.150628	0.137802	0.187312	0.223365	0.1963989
16	0.039754	0.034957	0.087974	0.136169	0.122115	0.0975869
18	0.334136	0.528552	0.433717	0.461528	0.598941	0.5912051
20	0.167313	0.224408	0.245873	0.228349	0.237817	0.170194
22	0.118215	0.138587	0.202067	0.165929	0.21327	0.1762547
24	0.139732	0.127649	0.108448	0.114207	0.099778	0.1353093

Table S6: Relative mRNA expression *bmal1* – incubation with strain *E. coli*, strain B17

***bmal1* BJ17**	Well A			Well B		
hours	Replicate 1	Replicate 2	Replicate 3	Replicate 1	Replicate 2	Replicate 3
2	0.172281	0.180982	0.183183	0.112157	0.095163	0.100034
4	0.137851	0.129667	0.135157	0.152739	0.1094	0.114819
6	0.097885	0.110564	0.097901	0.138959	0.140529	0.094102
8	0.207355	0.175667	0.134189	0.157159	0.194	0.236381
10	0.143606	0.144589	0.146259	0.250215	0.194632	0.200252
12	0.236476	0.139399	0.216863	0.191438	0.148943	0.131496
14	0.237434	0.19977	0.1984	0.168952	0.190003	0.127519
16	0.206604	0.197352	0.197727	0.171713	0.248062	0.137464
18	0.269173	0.275548	0.258878	0.344007	0.341382	0.391901
20	0.159083	0.201673	0.152717	0.220195	0.210558	0.238045
22	0.163982	0.122528	0.194323	0.155915	0.134428	0.126206
24	0.151058	0.158353	0.211478	0.221658	0.16641	0.183022

Table S7: Relative mRNA expression *bmal1* – incubation with strain *E. coli*, strain B23

***bmal1* BJ23**	Well A			Well B		
hours	Replicate 1	Replicate 2	Replicate 3	Replicate 1	Replicate 2	Replicate 3
2	0.157126	0.248348	0.19184	0.144265	0.153817	0.184905
4	1.387434	1.823275	1.017193	0.130479	0.150943	0.137079
6	0.088436	0.132437	0.126516	0.122031	0.115521	0.13119
8	0.130455	0.113814	0.152266	0.271343	0.249294	0.179769
10	0.140608	0.12404	0.172665	0.114261	0.14825	0.143474
12	0.255717	0.255407	0.251352	0.149649	0.161856	0.155424
14	0.171165	0.214971	0.213692	0.322502	0.188792	0.279216
16	0.315369	0.319591	0.255912	0.276875	0.470233	0.353451
18	0.303199	0.301418	0.369181	0.517213	0.490001	0.500535
20	0.116948	0.144515	0.241527	0.406596	0.371704	0.424165
22	0.1465	0.102355	0.133532	0.109508	0.171946	0.166292
24	0.122598	0.10717	0.106435	0.096113	0.137795	0.143457

Table S8: Relative mRNA expression *per1* – incubation with no bacteria, control

***per1* control**	Well A			Well B		
hours	Replicate 1	Replicate 2	Replicate 3	Replicate 1	Replicate 2	Replicate 3
2	0.002602	0.001808	0.002391	0.002063	0.002222	0.002645
4	0.002599	0.002056	0.001848	0.002029	0.001942	0.001506
6	0.0021	0.001509	0.001658	0.002846	0.001873	0.001706
8	0.002846	0.002024	0.002495	0.002406	0.002099	0.002216
10	0.002059	0.002391	0.00191	0.001776	0.002142	0.00274
12	0.001863	0.001992	0.001491	0.001649	0.00144	0.001115
14	0.003176	0.002177	0.002152	0.002374	0.001739	0.001956
16				0.00141	0.001483	0.001146
18	0.636065	0.623937	0.632584	1.076395	1.090551	1.089791
20	0.655219	0.641832	0.642699	0.001117	0.001022	0.001685
22	0.00192	0.001314	0.001589	0.001426	0.001136	0.001308
24	0.001229	0.001322	0.001131	0.001107	0.00112	0.002394

Table S9: Relative mRNA expression *per1* – incubation with *E. coli*, strain BJ17

***per1* BJ17**	Well A			Well B		
hours	Replicate 1	Replicate 2	Replicate 3	Replicate 1	Replicate 2	Replicate 3
2	0.001499	0.001712	0.001629	0.001934	0.001586	0.0015
4	0.002309	0.002191	0.002122	0.002021	0.001995	0.001749
6	0.001779	0.001497	0.001552	0.003667	0.003211	0.003502
8	0.001394	0.001443	0.001326	0.003383	0.002749	0.003051
10	0.001694	0.001762	0.002264	0.001398	0.00187	0.001445
12	0.001828	0.001628	0.001837	0.00144	0.001297	0.001127
14	0.001766	0.001994	0.001458	0.001657	0.001412	0.001093
16	0.001206	0.001129	0.001152	0.001547	0.001644	0.001822
18	0.615333	0.607345	0.614048	0.561111	0.559963	0.563045
20	0.001203	0.001464	0.001275	0.001239	0.001259	0.001208
22	0.001976	0.001426	0.001376	0.00099	0.000872	0.001109
24	0.000955	0.001175	0.001109	0.001987	0.002208	0.002561

Table S10: Relative mRNA expression *per1* – incubation with *E. coli*, strain BJ23

***per1* BJ23**	Well A			Well B		
hours	Replicate 1	Replicate 2	Replicate 3	Replicate 1	Replicate 2	Replicate 3
2	0.003864	0.003162	0.002626			
4	0.030303	0.044513	0.030729	0.001659	0.001224	0.0016
6	0.002536	0.001948	0.002228	0.00135	0.001326	0.001511
8	0.002002	0.002151	0.002207	0.002243	0.001835	0.002157
10	0.001685	0.002474	0.001622	0.00167	0.001253	0.001242
12	0.002027	0.001927	0.001692	0.002481	0.002389	0.002233
14	0.001919	0.001776	0.001891	0.001366	0.001386	0.000887
16	0.000728	0.000842	0.000944	0.002286	0.001279	0.001853
18	0.614476	0.606922	0.612792	0.660889	0.670456	0.689618
20				0.001145	0.000745	0.001373
22	0.000949	0.001022	0.00133	0.001207	0.001488	0.001071
24	0.001148	0.001259	0.001089	0.001254	0.00169	0.002302

Table S11: Relative mRNA expression *per2* – incubation with no bacteria, control

***per2* control**	Well A			Well B		
hours	Replicate 1	Replicate 2	Replicate 3	Replicate 1	Replicate 2	Replicate 3
2	0.350412	0.257638	0.287659	0.34555	0.354106	0.332937
4	0.216976	0.244292	0.197439	0.276782	0.251205	0.245602
6	0.231317	0.19433	0.189717	0.379789	0.307602	0.34712
8	0.174424	0.203311	0.167187	0.416969	0.363088	0.408583
10	0.232861	0.29715	0.327248	0.330204	0.327571	0.291097
12	0.319664	0.28061	0.265192	0.21108	0.247392	0.219628
14	0.243623	0.241334	0.261489	0.330575	0.267762	0.265683
16	0.288553	0.298702	0.119009	0.233162	0.19803	0.179666
18	0.195138	0.229565	0.191822	0.27117	0.240482	0.216805
20	0.213064	0.213817	0.190757	0.256132	0.230738	0.246059
22	0.222971	0.17123	0.1868	0.24281	0.248052	0.191816
24	0.214604	0.207004	0.239349	0.153566	0.235352	0.244641

Table S12: Relative mRNA expression *per2* – incubation with *E. coli*, strain BJ17

***per2* BJ17**	Well A			Well B		
hours	Replicate 1	Replicate 2	Replicate 3	Replicate 1	Replicate 2	Replicate 3
2	0.237322	0.246313	0.234688	0.389471	0.251775	0.371249
4	0.421644	0.371104	0.33866	0.34535	0.285966	0.311213
6	0.310454	0.29968	0.268584	0.341931	0.337536	0.349235
8	0.30527	0.26335	0.30158	0.433769	0.433373	0.412161
10	0.313138	0.235896	0.264438	0.244239	0.219636	0.235743
12	0.239351	0.228807	0.202916	0.203364	0.215336	0.211957
14	0.149996	0.218724	0.155798	0.187232	0.164351	0.206793
16	0.200455	0.346195	0.244301	0.222667	0.232108	0.225631
18	0.183945	0.186039	0.218579	0.174832	0.16531	0.163163
20	0.219141	0.204126	0.209104	0.175015	0.172489	0.202208
22	0.191882	0.175017	0.200284	0.202182	0.211508	0.218732
24	0.204348	0.178696	0.199076	0.357289	0.281522	0.277712

Table S13: Relative mRNA expression *per2* – incubation with *E. coli*, strain BJ23

***per2* BJ23**	Well A			Well B		
hours	Replicate 1	Replicate 2	Replicate 3	Replicate 1	Replicate 2	Replicate 3
2	0.418233	0.425247	0.492451	0.764761	0.758361	0.715877
4	5.118074	7.188135	5.591332	0.153604	0.182042	0.15752
6	0.417954	0.388305	0.382322	0.311365	0.298338	0.309443
8	0.431634	0.350232		0.207989	0.154659	0.201642
10	0.20206	0.218911	0.184533	0.190978	0.236092	0.192919
12	0.290318	0.272962	0.299498	0.277189	0.254664	0.272645
14	0.264472	0.32033	0.318423	0.250209	0.28132	0.225987
16	0.122115	0.17464	0.155913	0.200994	0.31557	0.305732
18	0.205949	0.202279	0.164269	0.114859	0.174343	0.159736
20	0.280896	0.34502	0.20766	0.21557	0.127544	0.176596
22	0.174562	0.189587	0.175263			
24	0.179401	0.161637	0.191277	0.219958	0.230312	0.253178

Table S14: Relative mRNA expression *per3* – incubation with no bacteria, control

***per3* control**	Well A			Well B		
hours	Replicate 1	Replicate 2	Replicate 3	Replicate 1	Replicate 2	Replicate 3
2	0.041451	0.040549	0.048466	0.083746	0.083746	0.076092
4	0.064405	0.057736	0.065145			
6	0.152288	0.076877	0.077508	0.061598	0.061598	0.114986
8	0.174933	0.094717	0.082969	0.127043	0.127043	0.123841
10	0.035339	0.083963	0.037383	0.130651	0.130651	0.107978
12	0.115906	0.075348	0.059822	0.052236	0.052236	0.035459
14						
16	0.108861	0.071335	0.070367	0.089283	0.089283	0.085076
18	0.074384	0.047532	0.037583	0.0553	0.0553	0.062795
20	0.071822	0.063388	0.142297	0.047302	0.047302	0.070233
22	0.053349	0.045249	0.087207	0.041519	0.041519	0.069586
24				0.054562	0.054562	0.069524

Table S15: Relative mRNA expression *per3* – incubation with *E. coli*, strain BJ17

***per3* BJ17**	Well A			Well B		
hours	Replicate 1	Replicate 2	Replicate 3	Replicate 1	Replicate 2	Replicate 3
2	0.07458	0.070993	0.084692			
4	0.115317	0.087851	0.134538	0.066686	0.078328	0.078959
6	0.057953	0.064758	0.087947	0.0492	0.128964	0.110034
8	0.070075	0.104997	0.06951	0.075534	0.112917	0.077009
10	0.081254	0.105734	0.111848	0.084555	0.068405	0.053465
12	0.076174	0.074012	0.047935	0.080084	0.062261	0.038952
14				0.067715	0.073117	0.093762
16	0.02836	0.029417	0.037642	0.039073	0.062296	0.031188
18				0.049988	0.047375	0.074307
20	0.032822	0.03519	0.056933	0.032056	0.046154	0.039226
22	0.081067	0.073078	0.078387	0.060978	0.062623	0.070301
24	0.052453	0.060149	0.087801	0.096128	0.116901	0.077232

Table S16: Relative mRNA expression *per3* – incubation with *E. coli*, strain BJ23

***per3* BJ23**	Well A			Well B		
hours	Replicate 1	Replicate 2	Replicate 3	Replicate 1	Replicate 2	Replicate 3
2	0.072096	0.05902	0.12614			
4	0.239206	1.076796	0.348067	0.049521	0.041726	0.016889
6	0.097104	0.067207	0.089394	0.059014	0.0698	0.093201
8	0.114328	0.079412	0.129397			
10	0.126219	0.114349	0.133511	0.094959	0.080117	0.073022
12	0.056818	0.069485	0.042524	0.033876	0.029084	0.067361
14	0.077753	0.076692	0.065331	0.127014	0.045335	0.110708
16	0.041142	0.059932	0.041999	0.111957	0.079159	0.073515
18	0.06404	0.056336	0.096742	0.052325	0.058905	0.036814
20	0.07768	0.030891	0.081516	0.060757	0.055562	0.066895
22	0.054397	0.067671	0.075712	0.033856	0.060394	0.050869
24	0.054858	0.075192	0.066212	0.080951	0.041007	0.100305

Table S17: Relative mRNA expression *cry1* – incubation with no bacteria, control

***cry1* control**	Well A			Well B		
hours	Replicate 1	Replicate 2	Replicate 3	Replicate 1	Replicate 2	Replicate 3
2	0.350822	0.26696	0.338822	0.297995	0.303341	0.305197
4	0.152523	0.214736	0.201216	0.27957	0.187275	0.17686
6	0.33171	0.228365	0.262786	0.305015	0.278515	0.272991
8	0.230289	0.279128	0.298782	0.232265	0.232854	0.225003
10	0.250128	0.310376	0.23463	0.323316	0.252998	0.260084
12	0.274708	0.260689	0.246916	0.23642	0.223272	0.250254
14	0.216007	0.234276	0.203309	0.247386	0.251386	0.218386
16	0.309263	0.336316	0.270802	0.257094	0.295757	0.281535
18	0.250392	0.203558	0.276872	0.321279	0.476495	0.314859
20	0.24193	0.226163	0.19577	0.203813	0.225196	0.212404
22	0.197373	0.180664	0.182085	0.172373	0.209018	0.236517
24	0.141589	0.193217	0.159221	0.170665	0.210278	0.184556

Table S18: Relative mRNA expression *cry1* – incubation with *E. coli*, strain BJ17

***cry1* BJ17**	Well A			Well B		
hours	Replicate 1	Replicate 2	Replicate 3	Replicate 1	Replicate 2	Replicate 3
2	0.209996	0.239082	0.229691	0.258866	0.268283	0.298059
4	0.211136	0.205408	0.201293	0.11475	0.200112	0.185939
6	0.237931	0.207913	0.207356	0.238833	0.29296	0.223151
8	0.220522	0.213999	0.221983	0.298375	0.209374	0.26723
10	0.24491	0.235922	0.272581	0.173485	0.2304	0.225721
12	0.196008	0.197971	0.219418	0.194486	0.218066	0.199696
14	0.184138	0.178655	0.184684	0.214856	0.184861	0.224382
16	0.179382	0.170588	0.205794	0.262442	0.222977	0.257366
18	0.293545	0.24022	0.244909	0.203905	0.263808	0.208748
20	0.208111	0.207308	0.201828	0.247752	0.260542	0.304745
22	0.226507	0.229972	0.209577	0.184266	0.218009	0.226171
24	0.177854	0.206937	0.200943	0.226984	0.200294	0.216398

Table S19: Relative mRNA expression *cry1* – incubation with *E. coli*, strain BJ23

***cry1* BJ23**	Well A			Well B		
hours	Replicate 1	Replicate 2	Replicate 3	Replicate 1	Replicate 2	Replicate 3
2	0.300021	0.336656	0.258498	1.325429	0.274471	0.326074
4	3.142712	4.639763	4.345631	0.192495	0.226541	0.194349
6	0.230762	0.254967	0.262719	0.198733	0.207293	0.193465
8	0.203609	0.182332	0.230425	0.247588	0.234673	0.295716
10	0.240869	0.225943	0.227739	0.22265	0.221712	0.247584
12	0.259613	0.237888	0.290907	0.215371	0.202777	0.189769
14	0.190085	0.198579	0.225461	0.289695	0.231546	0.256954
16	0.23572	0.243865	0.19355	0.209693	0.204903	0.322514
18	0.218685	0.217262	0.266352	0.209852	0.203934	0.240139
20	0.297727	0.326955	0.185321	0.16762	0.242703	0.204803
22	0.193292	0.183561	0.133219	0.151271	0.195994	0.180319
24	0.158244	0.147606	0.129125	0.20384	0.178161	0.194113

Table S20: Relative mRNA expression *cry2* – incubation with no bacteria, control

***cry2* control**	Well A			Well B		
hours	Replicate 1	Replicate 2	Replicate 3	Replicate 1	Replicate 2	Replicate 3
2	0.571484	0.564851	0.476913	0.49182	0.405912	0.526495
4	0.517	0.408531	0.395807	0.515433	0.444042	0.422949
6	0.636187	0.559175	0.55571	0.377296	0.560807	0.434739
8	0.526453	0.474245	0.503471	0.362798	0.361071	0.396516
10	0.54412	0.646968	0.487801	0.636636	0.529304	0.56155
12	0.495028	0.48511	0.472214	0.342263	0.358669	0.35455
14	0.459674	0.489329	0.351444	0.56713	0.484534	0.519105
16	0.443773	0.480936	0.52121	0.351631	0.379488	0.403786
18	0.304811	0.330988	0.314878	0.293368	0.244023	0.424322
20	0.355504	0.302732	0.360104	0.285092	0.281195	0.323385
22	0.374048	0.271296	0.297112	0.263168	0.283535	0.416696
24	0.252469	0.301427	0.274018			

Table S21: Relative mRNA expression *cry2* – incubation with *E. coli*, strain BJ17

***cry2* BJ17**	Well A			Well B		
hours	Replicate 1	Replicate 2	Replicate 3	Replicate 1	Replicate 2	Replicate 3
2	0.454196	0.418915	0.380765	0.533123	0.422942	0.577737
4	0.477771	0.399625	0.389377	0.333711	0.430596	0.37195
6	0.46115	0.382201	0.453084	0.551001	0.59366	0.616618
8	0.43797	0.442253	0.454987	0.615591	0.495495	0.557812
10	0.449664	0.404247	0.452429	0.403736	0.377535	0.353859
12	0.356665	0.393694	0.404553	0.350346	0.369331	0.363771
14	0.364286	0.307635	0.269002	0.497351	0.492497	0.317452
16	0.322861	0.319686	0.31204	0.371488	0.326508	0.321153
18	0.34254	0.461803	0.44109	0.330915	0.358653	0.329948
20	0.369223	0.389363	0.292358	0.352602	0.3844	0.337136
22	0.358507	0.347367	0.33906	0.311291	0.286283	0.234309
24	0.339433	0.256955	0.33089	0.395707	0.408344	0.417961

Table S22: Relative mRNA expression *cry2* – incubation with *E. coli*, strain BJ23

***cry2* BJ23**	Well A			Well B		
hours	Replicate 1	Replicate 2	Replicate 3	Replicate 1	Replicate 2	Replicate 3
2	0.575506	0.590416	0.551774	0.550604	0.633364	0.664927
4	6.31421	7.432243	5.560975	0.400728	0.452797	0.411873
6	0.397963	0.3457	0.406523	0.304072	0.34346	0.321954
8	0.328132	0.308774	0.33778	0.541804	0.521422	0.504282
10	0.427272	0.468121	0.400368	0.36774	0.328427	0.327492
12	0.489548	0.425195	0.480572	0.420651	0.351923	0.359105
14	0.455003	0.428634	0.423243	0.467251	0.462588	0.381364
16	0.343661	0.395674	0.367322	0.353744	0.37236	0.370892
18	0.34652	0.30709	0.237344	0.45087	0.324653	0.246117
20	0.372318	0.290509	0.342773	0.34132	0.25516	0.262914
22	0.298126	0.309702	0.307737	0.363982	0.338916	0.329334
24	0.277729	0.270156	0.25661	0.313233	0.324874	0.313148

Table S23. Comparison of circadian behaviour of core clock gene expression in HepG2 cells (K) in co-culture with *E. coli* strains BJ17 and BJ23, related to Figure 2. The statistically significant changes are shown in bold.

Gene, comparison	Model significance, adjusted p-value	Amplitude change (a.u.)	Amplitude change, adjusted p-value	Phase shift (radians)	Phase shift (hours)	Phase shift, adjusted p-value
CLOCK, BJ17:K	1.16E-01	4.18E-02	1.89E-01	-0.05	0.20	9.74E-01
CLOCK, BJ23:K	5.16E-02	6.08E-02	1.17E-01	-0.50	1.90	9.74E-01
BMA1L, BJ17:K	2.65E-03	2.40E-02	1.74E-01	-0.11	0.44	9.74E-01
BMAL1, BJ23:K	1.08E-03	5.67E-02	9.84E-02	-0.23	0.87	9.74E-01
PER1, BJ17:K	6.14E-07	-1.28E-01	**2.66E-04**	3.19	-12.17	9.74E-01
PER1, BJ23:K	7.73E-05	-8.54E-02	8.53E-02	0.05	-0.18	9.74E-01
PER2, BJ17:K	3.99E-12	3.81E-02	**1.93E-02**	0.57	-2.19	1.48E-01
PER2, BJ23:K	2.20E-04	4.19E-02	1.39E-01	0.95	-3.62	1.48E-01
PER3, BJ17:K	8.77E-06	7.50E-03	2.55E-01	1.11	-4.23	**4.07E-02**
PER3, BJ23:K	1.11E-02	7.98E-03	4.37E-01	0.88	-3.37	2.69E-01
CRY1, BJ17:K	1.31E-03	-1.65E-02	1.50E-01	2.29	-8.77	1.48E-01
CRY1, BJ23:K	5.19E-01	6.52E-03	7.96E-01	1.84	-7.03	1.48E-01
CRY2, BJ17:K	5.81E-13	-2.60E-02	1.89E-01	0.50	-1.90	1.48E-01
CRY2, BJ23:K	2.30E-09	-4.55E-02	9.23E-02	0.38	-1.46	4.30E-01

Table S24. Final dilutions of the bacterial culture in growth curve experiments, related to Bacterial Growth Curves.

Time point	Final dilution on LB plates
0	$10^{-3} – 10^{-5}$
1	$10^{-3} – 10^{-6}$
2	$10^{-4} – 10^{-6}$
3	$10^{-5} – 10^{-7}$
4	$10^{-5} – 10^{-8}$
5	$10^{-6} – 10^{-8}$
6	$10^{-6} – 10^{-8}$
7	$10^{-6} – 10^{-8}$

Table S25. List of used primers for RT-PCR, related to RNA isolation and RT-PCR analysis.

Primer	Primer sequence (5' → 3')	Reference
CLOCKfw	TGCGAGGAACAATAGACCCAA	(Luo et al., 2016)
CLOCKrev	ATGGCCTATGTGTGCGTTGTA	(Luo et al., 2016)
BMAL1fw	AAGGGAAGCTCACAGTCAGAT	(Luo et al., 2016)
BMAL1rev	GGACATTGCGTTGCATGTTGG	(Luo et al., 2016)
CRY1fw	CTCCTCCAATGTGGGCATCAA	(Luo et al., 2016)
CRY1rev	CCACGAATCACAAACAGACGG	(Luo et al., 2016)
CRY2fw	TCCCAAGGCTGTTCAAGGAAT	This study
CRY2rev	TGCATCCCGTTCTTTCCCAAA	This study
PER1fw	CTACGGCATGTGCACAGTAGA	This study
PER1rev	GTGTGTACTCAGACGTGATGTG	This study
PER2fw	GACATGAGACCAACGAAAACTGC	(Luo et al., 2016)
PER2rev	AGGCTAAAGGTATCTGGACTCTG	(Luo et al., 2016)
PER3fw	GCAGAGGAAATTGGCGGACA	(Luo et al., 2016)
PER3rev	GGTTTATTGCGTCTCTCCGAG	(Luo et al., 2016)
*RPLP0fw**	TGCATCAGTACCCCATTCTATCA	This study
*RPLP0rev**	AAG?TGTAATCCGTCTCCACAGA	This study
*β-AKTINfw**	TCCTCTCCCAAGTCCACACAGG	This study
*β-AKTINrev**	GGGCACGAAGGCTCATCATTC	This study
IL6Rfw	CCCCTCAGCAATGTTGTTTGT	(Wu et al., 2017)
IL6Rrev	CTCCGGGACTGCAACTGG	(Wu et al., 2017)
TNFfw	CCTCTCTCTAATCAGCCCTCTG	(Li et al., 2016)
TNFrev	GAGGACCTGGGAGTAGATGAG	(Li et al., 2016)

* For housekeeping genes

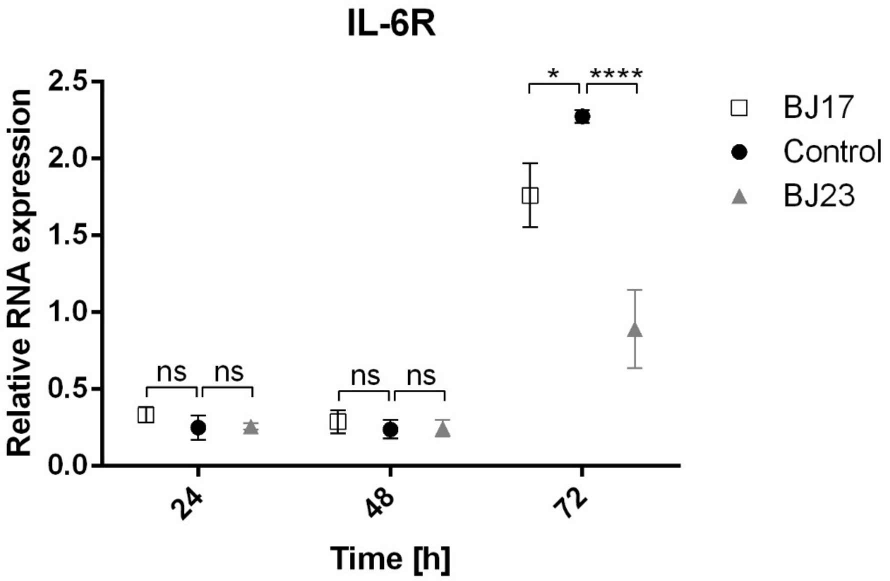

Figure S2: Relative expression of the IL-6R genes as a function of time (24–72 hours). BJ17 (white squares), BJ23 (grey triangles) – co-cultures of BJ strain with the HepG2 cells. Control (black circles) – HepG2 cells without bacteria. Statistically significant results were presented; not significant (ns) for P > 0.05, * for P ≤ 0.05, ** for P ≤ 0.01, *** for P ≤ 0.001 and **** for P ≤ 0.0001. Data are represented as mean ± SD of two separate experiments.

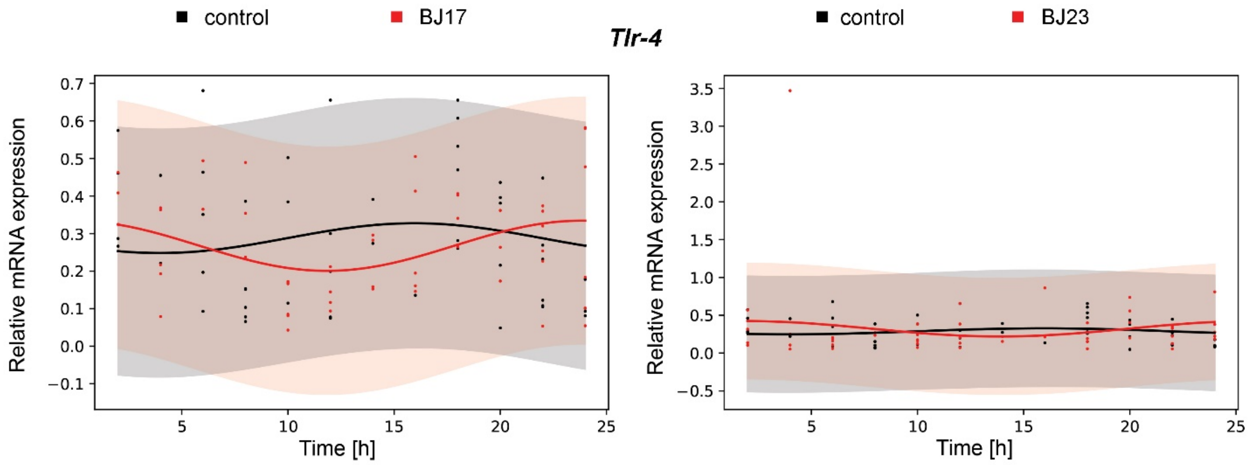

Figure S3: Comparison of circadian behaviour of core clock gene expression between the co-cultures and control. The figures represent the relative RNA expression of the TLR4 genes as a function of time (2–24 hours) in HepG2 cells (K, black dots and lines, respectively) and in HepG2 cell co-**cultures** with *E. coli* strains BJ17 and BJ23 (red dots and lines, respectively). The experimental results, obtained in two separate experiments are represented with dots, and the fitted cosinor curves with solid lines.

References in Supplementary Files

Li, X., Wang, S., Zhu, R., Li, H., Han, Q., Zhao, R.C., 2016. Lung tumor exosomes induce a pro-inflammatory phenotype in mesenchymal stem cells via NFκB-TLR signaling pathway. J. Hematol. Oncol. 9, 1–12. https://doi.org/10.1186/s13045-016-0269-y

Luo, W., Ma, S., Yang, Y., Wang, C., Zhang, D., Zhang, Q., Liu, Y., Liu, Z., 2016. TFEB regulates PER3 expression via glucose-dependent effects on CLOCK/BMAL1. Int. J. Biochem. Cell Biol. 78, 31–42. https://doi.org/10.1016/j.biocel.2016.06.020

Wu, Y.S., Chung, I., Wong, W.F., Masamune, A., Sim, M.S., Looi, C.Y., 2017. Paracrine IL-6 signaling mediates the effects of pancreatic stellate cells on epithelial-mesenchymal transition via Stat3/Nrf2 pathway in pancreatic cancer cells. Biochim. Biophys. Acta - Gen. Subj. 1861, 296–306. https://doi.org/10.1016/j.bbagen.2016.10.006
